# Changes in plant C, N and P ratios under elevated [CO_2_] and canopy warming in a rice-winter wheat rotation system

**DOI:** 10.1038/s41598-019-41944-1

**Published:** 2019-04-01

**Authors:** Jianqing Wang, Xiaoyu Liu, Xuhui Zhang, Lianqing Li, Shu Kee Lam, Genxing Pan

**Affiliations:** 1 Institute of Resource, Ecosystem and Environment of Agriculture, and Center of Climate Change and Agriculture, Nanjing Agricultural University, 1 Weigang, Nanjing, 210095 China; 2 Key Laboratory for Humid Subtropical Eco-geographical Processes of the Ministry of Education, Fujian Normal University, Fuzhou, 350007 China; 3 Tohoku Agricultural Research Center, National Agricultural and Food Research Organization, Iwate, 020-0198 Japan; 4 School of Agriculture and Food, Faculty of Veterinary and Agricultural Sciences, The University of Melbourne, Parkville, VIC 3010 Australia

## Abstract

Elevated atmospheric CO_2_ concentration ([CO_2_]) can stimulate plant growth through enhanced photosynthetic rate. However, plant C, N and P ratios in response to elevated [CO_2_] combined with canopy warming in rice-winter wheat rotation system remain largely unknown. Here we investigated the impacts of elevated [CO_2_] and warming on plant nutrient ratios under open-air conditions. Four treatments including the ambient condition (CK), elevated [CO_2_] (500 ppm, CE), canopy warming (+2 °C, WA), and the combination of elevated [CO_2_] and warming (CW) were used to investigate the responses of plant C, N and P ratios in a rice-winter wheat rotation system in southeast China. Results showed that elevated [CO_2_] increased C:N ratio in whole plant by 8.4–14.3% for both crops, and increased C:P ratio by 11.3% for rice. The changes in ratio were due to an increase in C concentration by 0.8–1.2% and a reduction in N concentration by 7.4–10.7% for both crops, and a reduction in P concentration by 10.0% for rice. Warming increased N allocation in rice leaf and N concentration by 12.4% for rice, resulting in increases in the ratios of N to C and P by 11.9% and 9.7% in rice, but not in wheat. However, CW had no effect on plant C:N ratio in rice, indicating the positive effect of elevated [CO_2_] could offset the negative impact of warming on C:N ratio. By contrast, CW significantly decreased plant C:P and N:P ratios by 16% due to the increase in P allocation in stem for wheat. These results suggest that impacts of climate change on plant nutrient balance occur through interactions between the effects of climate change on nutrient uptake and allocation, which is important for food quality and productivity under global climate change.

## Introduction

Global atmospheric carbon dioxide concentration ([CO_2_]) has increased rapidly due to the ongoing anthropogenic activities since the industrial revolution^1^. It is a common belief that elevated [CO_2_] stimulates terrestrial plant growth by enhancing CO_2_ fixation rate, known as the [CO_2_] fertilization effect^2–4^. Elevated [CO_2_] also benefits plant growth in drought regions by promoting water use efficiency via reduction in stomatal conductance^5,6^. However, plant growth promotion might be constrained by the nutrient deficiency under elevated [CO_2_], as the plants demand more nutrients for growth under elevated [CO_2_]^7^. Previous studies suggested that future plants would be exposed to a global nutrient imbalance under elevated [CO_2_]^8–11^, with lower N concentration^10^ or increased C:N and C:P ratios in plant functional organs^9^. However, the response of C:N or C:P ratio to elevated [CO_2_] showed large variation across various ecosystems^11^. This might be resulted from the different nutrient management regimes across these systems. In forest or grassland ecosystems, progressive occurrence of nutrient limitation under elevated [CO_2_] may occur as fertilization are seldom performed^7^. In contrast, in agroecosystems nutrient limitation may not occur under elevated [CO_2_] due to sufficient nutrient supply from fertilizers. Nevertheless, some studies reported that elevated [CO_2_] increased C:N ratio^12,13^. So far, it remains unclear how elevated [CO_2_] impact the direction and magnitude of nutrient uptake and their ratios in agroecosystems.

It is increasingly important to recognize that the responses of plants to elevated [CO_2_] are associated with other factors that change terrestrial ecological processes, in particular global warming. Global average surface temperature is estimated to rise by 1.1–6.4 °C at the end of 21^st^ century^14^. Unlike elevated [CO_2_], global warming can either decrease plant productivity by shortening plant growing period and reducing panicle number in warm regions^15–18^ or stimulate plant grown in cold regions^19,20^. Under a combination of elevated [CO_2_] and warming, the overall nutrient uptake and their ratio to carbon in plants can also be changed. Some studies have reported that warming increase nutrient concentrations in plants^21,22^. However, Yuan and Chen^11^ reported that warming alone had no effect on plant N concentration but decreased plant P concentration across a range of plant communities. In comparison, the combination of elevated [CO_2_] and warming had a neutral effect on N and P concentrations and N:P ratio in plants^11,23^.

In addition to nutrient concentrations and their ratios in the whole plants, it is increasingly important to understand the nutrient allocation across different plant functional organs. In some studies, nutrient concentrations of the whole plant were not affected by elevated [CO_2_] or warming. However, being unresponsive in the whole plant does not necessarily mean that the nutrient concentrations in a specific functional organ is not affected. Inconsistent responses of plant nutrient concentrations across different functional organs have been reported^13,22,24,25^. For instance, Cheng *et al*. found that elevated night (from 20:00 until 04:00) temperature by ca 10 °C had no effect on whole rice plant C or N concentration, but increased N concentration in the living leaf and reduced its allocation to the ear^13^. Results from these studies were mainly dependent on plant types and experimental conditions. To date, the effect of elevated [CO_2_] and canopy warming on plant nutrient (C, N and P) ratios in a rice-wheat rotation system is not well understood.

The main objective of this study was to examine the changes in plant nutrient (C, N and P) uptake and their ratios in different plant functional organs in a summer rice-winter wheat rotation system under simulated climate change conditions. We hypothesized that elevated [CO_2_] had a positive effect on C to N and C to P ratios due to dilution effect; warming would increase nutrient concentrations, and alter plant C, N and P ratios under elevated [CO_2_] and warming. We aim to provide knowledge for improving food quality and nutrient management in agriculture under future climate change.

## Results

### C, N and P concentrations in the whole plant

**Figure 1:**
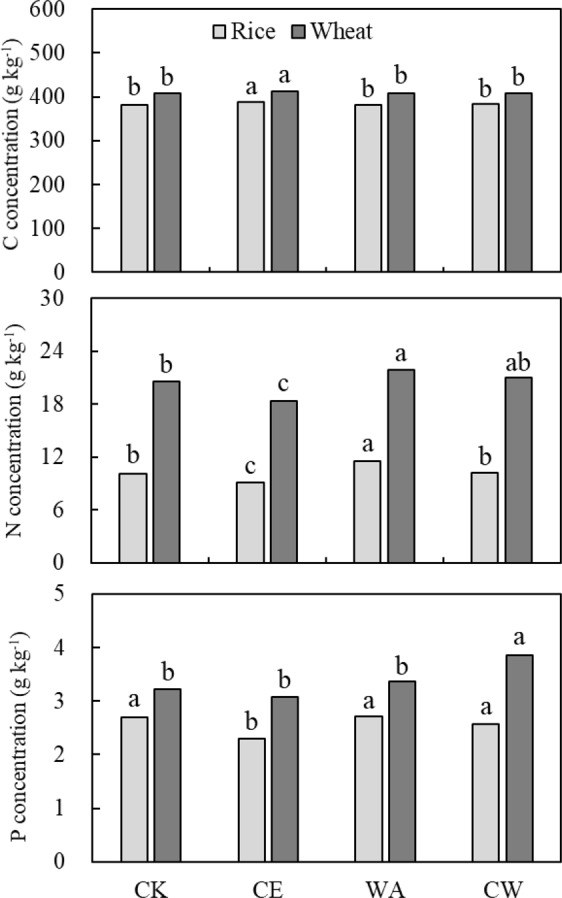
Changes in C, N and P concentrations in whole plant for rice (gray histogram) and wheat (black histogram) across three growth stages under simulated climate change conditions. Different letters indicate significant differences between treatments at *p* < 0.05.

Average cross three crop growth stages, elevated [CO_2_] increased C concentration by 1.2% and 0.8% for rice (*p* = 0.001) and wheat (*p* = 0.061), respectively; whereas warming decreased C concentration by 0.8% for rice (*p* < 0.05) (Fig. 1). A significant interaction between [CO_2_] and warming was observed for C concentration in rice (*p* < 0.05). Elevated [CO_2_] decreased N concentration by 10.7% and 7.4% for rice and wheat (*p* < 0.001); and warming increased that by 12.4% and 10.5% for rice and wheat (*p* < 0.001), respectively. Elevated [CO_2_] decreased P concentration by 10.0% in rice (*p* < 0.05), but not in wheat (*p* = 0.156). In contrast, warming increased P concentration by 14.8% in wheat (*p* < 0.001), but not in rice (*p* = 0.186).

The responses of C concentration to elevated [CO_2_] in different organs were similar to the whole plant (Tables S1, S2). For wheat, all treatments increased C concentration in stem and leaf at the ripening stage. However, the responses of N concentration to elevated [CO_2_] and warming varied with plant organs. Elevated [CO_2_] decreased N concentration by 21.5% and 7.1% in leaf for rice and wheat. Warming increased N concentration by 21.8% in leaf for rice, but decreased that for wheat. However, warming increased N concentration by 6.1% and 6.7% for rice (*p* < 0.01) and wheat (*p* = 0.075), respectively. The responses of P concentration to treatments also varied with organs. Elevated [CO_2_] increased P concentration by 11.8% and 10.5% in panicle/spike for rice (*p* = 0.010) and wheat (*p* = 0.056), while decreased that in stem and leaf of rice, and had no effect for wheat. However, warming increased P concentration in rice panicle and in wheat stem, but had no significant effect in other organs.

### Plant C, N and P ratios

**Figure 2:**
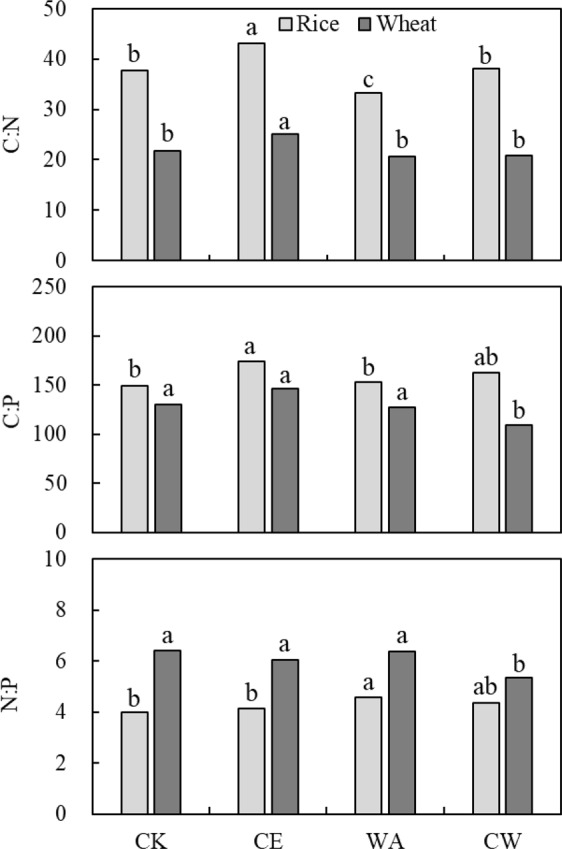
Changes in C:N, C:P and N:P ratios in whole plant for rice (gray histogram) and wheat (black histogram) across three growth stages under simulated climate change conditions. Different letters indicate significant differences between treatments at *p* < 0.05.

Plant C, N and P ratios in the whole plant exerted some remarkable changes under the treatments for both crops (Tables S3, S4). Generally, elevated [CO_2_] increased C:N ratio by 14.3% and 8.4% (*p* < 0.001), while warming reduced C:N ratio by 11.9% and 11.4% for rice and wheat (*p* < 0.001), respectively (Tables S3, S4). However, CW had no effect on C:N ratio in the whole plant for both crops (Fig. 2). Elevated [CO_2_] increased C:P ratio by 11.3% in the whole rice (*p* = 0.015), while warming had no effect on that. In contrast, warming decreased C:P ratio by 14.4% in the whole wheat, but the ratio was not affected by elevated [CO_2_]. Warming increased the N:P ratio by 9.7% in the whole rice (*p* < 0.05). Elevated [CO_2_] decreased the N:P ratio by 10.9% in the whole wheat (*p* < 0.01). Interaction effects between crop growth stages and treatments were observed in this study (Tables S3, S4). For example, elevated [CO_2_] significantly increased C:P ratio at the elongation and heading stages of rice, but not at the ripening stage.

The responses of C, N and P ratios across plant stem, leaf and panicle/spike varied between rice and wheat (Tables S3 and S4). For rice, leaf and panicle were more sensitive to elevated [CO_2_] and warming than stem; while stem was more responsive than leaf and spike for wheat. More specifically, elevated [CO_2_] increased the C:N ratio by 29.3% in rice leaf while warming alone decreased that by 20.1% (*p* < 0.001) (Table S3). For rice panicle, elevated [CO_2_] and warming generally decreased the C:N, C:P and N:P ratios. For wheat, elevated [CO_2_] had no significant effect on C, N and P ratios in different organs. However, warming decreased C:N and N:P ratios in stem, but increased C:N and C:P ratios in leaf for wheat.

### C, N and P allocations across organs

**Figure 3:**
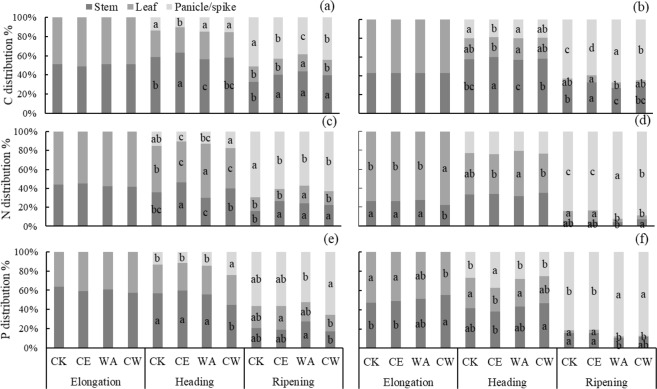
Effects of elevated [CO_2_] and warming on the amount of C (above panel), N (middle panel) and P (below panel) distribution in stem, leaf and panicle (pike) for rice (**a**,**c**,**e**) and wheat (**b**,**d**,**f**). Different letters indicate significant differences between treatments at *p* < 0.05.

At the elongation stage, elevated [CO_2_], warming or their combination had no effect on C allocation across leaf or stem (Fig. 3a,b). However, at the heading and ripening stages, elevated [CO_2_] and warming significantly affected C allocation. Elevated [CO_2_] reduced the C allocation from stem to panicle/spike for both crops, but had no significant effect on leaf. At the ripening stage, warming alone generally decreased C allocation in panicle/spike for rice, and increased it for wheat. Additionally, warming increased the allocation of C in rice leaf. CW increased C allocation in rice panicle.

The responses of N allocation to elevated [CO_2_] and warming differed between rice and wheat (Fig. 3c,d). Elevated [CO_2_] decreased N allocation in panicle at the heading and ripening stages in rice, but not in wheat. Warming increased N allocation in rice stem and leaf at the ripening stage. For wheat, warming decreased N allocation in leaf, but increased it in panicle/spike at the ripening stage. CW decreased N allocation in the panicle of rice, but increased it in the panicle/spike of wheat.

Treatment effects on P allocation varied across growth stages for both crops (Fig. 3e,f). For rice, none of the treatments had significant effect on P allocation except for a significant increase in plant panicle under CW at the heading stage. For wheat, CW decreased P allocation in leaf at the elongation and ripening stages (Fig. 3e). Elevated [CO_2_] decreased P allocation in leaf but increased P allocation in spike at the heading stage. Warming alone increased P allocation in spike but decreased it in leaf and stem at the ripening stage.

### Relation between C and nutrient (N and P)

**Figure 4:**
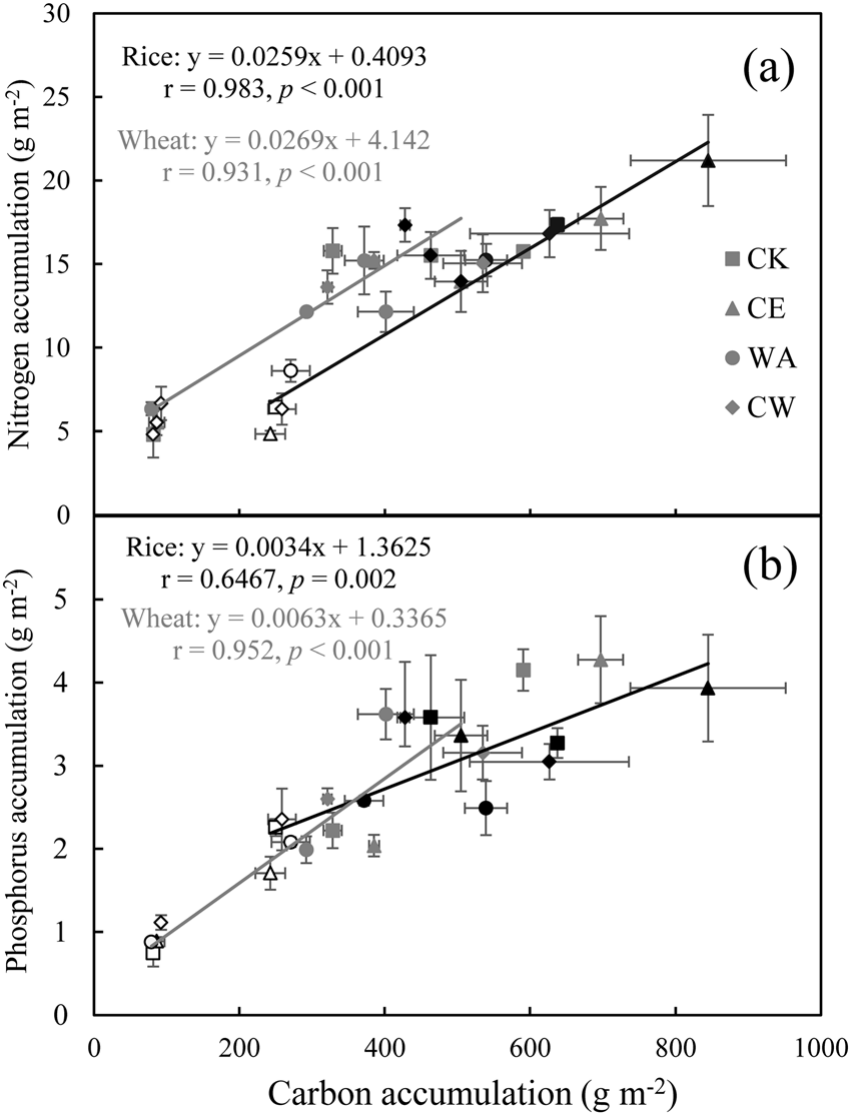
Relationships between C accumulation and N accumulation (**a**), P accumulation (**b**) for rice (black regressions lines) and wheat (gray regressions lines) during elongation (white), heading (gray) and ripening (black) stages under simulated climate change conditions.

The N and P accumulation were positively correlated with C accumulation for both crops (Fig. 4). Moreover, a negative correlation was also found between the C concentration and nutrient concentrations in stems for both crops (*p* < 0.05, Table 1).

**Table 1:** Pearson correlation coefficient between C concentration and N, P concentration in stem (n = 12), leaf (n = 12), panicle/spike (n = 8) and whole plant (n = 12) for rice and wheat.

C concentration		N concentration		P concentration	
		r	*p*	r	*p*
Rice	Stem	−0.631	0.028	−0.825	0.001
	Leaf	0.160	0.696	0.176	0.584
	Panicle/spike	0.193	0.648	−0.367	0.371
	Whole plant	−0.545	0.067	0.008	0.980
Wheat	Stem	−0.833	0.001	−0.799	0.002
	Leaf	−0.341	0.278	−0.275	0.387
	Panicle/spike	0.526	0.181	0.346	0.402
	Whole plant	−0.013	0.968	−0.046	0.887

## Discussion

Crop biomass and yield response and accordingly water and nutrient use efficiency, an issue of food supply and nutrition, had been already addressed in previous studies^6,16,26–28^. Our previous study shown that biomass ranged from 990 g m^−2^ to 1410 g m^−2^ for wheat, and from 1337 g m^−2^ to 1789 g m^−2^ for rice across the treatments^16^. Rice biomass was significantly greater than wheat. Elevated [CO_2_] increased crop biomass by 17.6% for both crops, while warming decreased biomass by 17.2% for wheat and 12.1% for rice. There was no significant effect between combined treatment and ambient condition. A similar trend was also observed in grain yield for both crops. However, the present study addresses how elevated [CO_2_] and warming impact on nutrients uptake and their ratios in different plant functional organs, an issue related to crop productivity and food nutrition value.

### Effect of elevated [CO_2_] on plant C, N and P ratios

Our findings partially supported the hypothesis that elevated [CO_2_] increased C concentration, but reduced N and P concentrations for both crops. An increase in C:N and C:P ratios (except for wheat) was accordingly observed under elevated [CO_2_]. The decrease in N and P concentrations under elevated [CO_2_] may result from a dilution effect due to biomass increase (13.5–17.7%) under elevated [CO_2_] for both crops^16^. There was a negative correlation between C concentration and nutrient (N and P) concentrations in stem (Table 1). This is in agreement with previous studies^9,11,29^ conducted across various ecosystems. However, Yuan and Chen^11^ found that elevated [CO_2_] had no effect on C:N and C:P ratios in deciduous and evergreen woody angiosperms. This indicated that the responses of C:N and C:P ratios to elevated [CO_2_] varied greatly among plants.

To date, few experiments have tested the response of nutrient ratios to elevated [CO_2_] across different plant functional organs. We expected that different plant organs have the same responses to elevated [CO_2_]. However, our results showed that elevated [CO_2_] induced greater decrease in nutrient concentration in leaf than in stem and panicle/spike (Tables S1, S2). Elevated [CO_2_] generally increased leaf C:N and C:P ratios but had no effect on stem and panicle/spike. This is due to the different nutrient translocation patterns with crop growth. Therefore, elevated [CO_2_] induced plant nutrient imbalance, particularly C, N and P balance under future climates^11^.

### Effect of warming on plant C, N and P ratios

Our results showed that warming alone decreased C:N ratio of rice because of the increased N concentration in rice and unchanged C concentration. Cheng *et al*. also observed that elevated night temperature had no effect on C concentration in rice despite an increase in plant respiration^13^. Additionally, An *et al*. reported that warming enhanced plant N concentration by 29.8–32.7% in a grassland ecosystem^30^. The change in N concentration could be ascribed to increased leaf transpiration rate under warming condition^21^. Warming increase plant leaf transpiration rate, leading to higher water requirement, which drives nutrient translocation from belowground to aboveground^31,32^. This is further confirmed by our observation that warming stimulated N transport from belowground. These results imply that global warming would exert a stronger effect on N uptake than on C assimilation, resulting in an imbalance in plant C and N content.

Additionally, warming alone had no effect on P concentration in the whole plant in this study (Fig. 1c), which led to an increase in N:P ratio in rice (Fig. 2). This suggests differential responses of N and P concentrations to warming alone. This might be explained by three possible reasons. Firstly, we inferred that the demand of N was more than that of P, as N is an important organ of organic compounds (e.g. amino acids, amides, proteins, nucleic acids, nucleotides, coenzymes, chlorophyll) for plant metabolism; whereas P is a component of sugar phosphates, which is less than N in plant functional organs^33^. Secondly, high temperature may have kinetic effects on the photosynthetic and respiration rate^34,35^, which requires more N input. Thirdly, our previous study has reported that warming had different effects on soil micronutrient availability through changes in soil environmental conditions (e.g. soil pH, moisture and microbial biomass)^32^. The increase of N:P ratio can be attributed to increases in soil nitrification rate and net N mineralization^36^, but reduction in soil P availability^37^. Therefore, warming increased N uptake from below ground, and influenced nutrient ratios in plant. As mentioned above, increment of N uptake in rice was higher than that of P. This is consistent with the study conducted by Reich and Oleksyn^38^, who also found that the N:P ratio increased with increment of air temperature.

Interestingly, different from rice, warming did not alter ratios of C, N and P in whole wheat (Fig. 2). Our results here demonstrated that warming did not alter N allocation and concentration in leaf for wheat, while a significant increase in N allocation to leaf was observed in rice (Fig. 3 and Tables S1, S2). Winter wheat was more sensitive than rice to warming^17,39^, resulting in limitation in wheat growth and N uptake under warming conditions. Furthermore, the N concentration increment of 13.4% in rice was higher than wheat (6.7%) under warming. This may be attributed to different N fertilizer input to rice (280.5 kg N ha^−1^) *vs* wheat (112.5 kg N ha^−1^). Therefore, the response in nutrient ratios to global warming would depend on crop types and agronomic management.

### Effect of elevated [CO_2_] and warming on plant C, N and P ratios

Numerous studies have reported that elevated [CO_2_] altered nutrient balance by increasing carbohydrate production^40–43^, but few studies have considered the combined effects of elevated [CO_2_] and warming^8,13,24^. Our results revealed that the responses of plant nutrient ratios to a combination of elevated [CO_2_] and warming were remarkably differed from that to elevated [CO_2_] or warming alone (Fig. 2), because of the offset effects between elevated [CO_2_] and warming^32,44^. For example, C:N ratio was significantly increased by elevated [CO_2_] alone, decreased by warming alone (Fig. 2a), but was unaffected under the combined treatment for either crop. This indicates that the positive effect of elevated [CO_2_] on C:N ratio compensate for the negative impact of warming. This is similar to the changes in crop productivity under the combined effects of elevated [CO_2_] and warming. This indicates that the effects of elevated [CO_2_] offset the impacts of warming on crop growing and nutrient uptake^16,17^. Our study demonstrated that the combination of elevated [CO_2_] and warming had no effect on C or N concentration, but remarkably increased P concentration in wheat (Fig. 1). This was attributed to the responses of nutrient uptake and allocation to the combined treatment of elevated [CO_2_] and warming, which significantly increased P allocation and concentration in stem for wheat (Fig. 3f and Tables S1, S2). This was in agreement with Bhattacharyya *et al*.’s study, which showed that combined elevated [CO_2_] and warming significantly increased crop P uptake^45^. This is due to the increase in organic acid from root exudate and P mineralization under the combination of elevated [CO_2_] and warming, which solubilized P in soil^42,45^. This would lead to an imbalance of P with other elements in winter wheat field. Our previous study has found that the rice biomass was higher than wheat^16^, resulting in more P demand for rice. The mechanisms of nutrient cycling under combined effects of elevated [CO_2_] and warming may be more complex than elevated [CO_2_] or warming alone. This information is important for the sustainability of nutrient availability in agroecosystems under future climate change.

## Conclusions

This study demonstrated that elevated [CO_2_] or warming alone significantly affected plant nutrient ratios in an agroecosystem, which varied with plant types and functional organs. Averaged across three key growth stages, elevated [CO_2_] increased C:N ratio for both crops mainly by reducing N concentration, whereas warming decreased C:N ratio while increased N:P ratio in rice due to enhanced N uptake and allocation of N in leaf. The combination of elevated [CO_2_] and warming had no effect on C:N ratio, but decreased C:P and N:P ratios in wheat. This was attributed to the increase in P allocation in wheat stem. The responses of nutrient uptake and ratios under combined elevated [CO_2_] and warming were different from that under elevated [CO_2_] or warming alone. This suggests that a offset effect exists between elevated [CO_2_] and warming. Therefore, the impact of climate change (elevated [CO_2_] and warming) on crop nutrient dynamics would be better predicted by a combination of these two factors rather than elevated [CO_2_] or warming alone.

## Materials and Methods

### Site description

This study was conducted in a field experiment station, where simulated elevated atmospheric [CO_2_], warming and their combination were performed to investigate the effect of climate change on an agroecosystem. The experimental site was established in 2010 and located at Kangbo village (31°30′N, 120°33′E), Guli Township, Changshu Municipality in Jiangsu Province, China. This area has a subtropical monsoon climate with a mean annual precipitation of 1100–1200 mm and annual average temperature of 16 °C over the last decade. The study area was a typical paddy field in the Taihu Lake region. The soil was a Gleyic Stagnic Anthrosol formed on clayey lacustrine deposit with a loamy texture (34% sand, 39% silt and 26% clay). The topsoil (0–20 cm) had 19.4 g kg^−1^ total C, 1.3 g kg^−1^ total N, 0.9 g kg^−1^ total P and a soil pH (H_2_O) of 7.0^27^.

### Experimental design

The experimental system was constructed under the state project of “Climate Change Impacts on Crop Production and Mitigation”, developed and managed by the Institute of Resource, Ecosystem and Environment of Agriculture (IREEA), Nanjing Agricultural University. The operational procedures of the facility were described in the work by Wang *et al*.^16^. In brief, the facility was designed to investigate two factors of climatic change, including elevated [CO_2_] of up to 500 ppm (CE), warming of canopy air by 2 °C with infrared heater over the crop canopy (WA), and a combination of these two treatments (CW), with ambient [CO_2_] without warming being the control (CK). Each treatment was deployed in an octagonal ring with a diameter of 8 m (area of ca 50 m^2^) and with three replications, totalling 12 rings. For elevated [CO_2_] treatments, pure CO_2_ gas via a liquid tank was injected into the ring plot with perforated pipes surrounding the ring. CO_2_ gas release was automatically manipulated based on ambient [CO_2_] and wind direction and speed. A total of 17 CO_2_ gas monitoring points were evenly distributed in each ring to determine the spatial variation of atmospheric [CO_2_]. Canopy air warming was performed with infrared heaters, hanging over the ring plot. A total of 12 infrared heaters (IR) (2000 W, 240 V, 1.65 m long × 0.14 m wide; HS-2420, Kalglo Electronics Co., Inc., Bethlehem, PA, USA) were equipped for each ring plot. The IR lamps produced invisible radiation to elevate the canopy air temperature. Both elevated [CO_2_] and canopy warming occurred throughout the crop growing period (rice, June-November; wheat, November-May). All the rings were separated by the adjacent open fields with about 28 m apart to avoid any treatment cross-contamination. The average [CO_2_] were 514 ± 55 ppm in rice season of 2012, and 505 ± 26 ppm in wheat season of 2012–2013 under the elevated [CO_2_] treatments, while the increment of canopy air temperature was a mean of 1.8 ± 0.6 °C in rice season of 2012, and 1.5 ± 0.7 °C in wheat season of 2012–2013 in the warming treatments, respectively. The performance and maintenance of these treatments were carefully managed throughout the crop growing periods.

### Agronomic management

Rice (*Oryza sativa* L. cv. Changyou No. 5) seedlings were transplanted on 15^th^ June 2012 and harvested on 28^th^ October 2012. Winter wheat (*Triticum aestivum* L. cv. Yangmai No.14) was sown at a density of 250 seedlings m^−2^ with a row spacing of 20 cm on 23^rd^ November 2012, and harvested on 2^nd^ June 2013. Field management, including weed and pest control, fertilization and irrigation, was carried out following the local farmers’ practice. Fertilizers were applied four times during rice season and three times during wheat season. Urea (46% N) was applied as basal fertilizers at a rate of 188 kg ha^−1^, and as topdressing fertilizer at a rate of 150 kg ha^−1^ once in the wheat seasons and twice in the rice seasons. Compound fertilizer (N: P_2_O_5_: K_2_O ratio, 15:15:15) was applied at 375 kg ha^−1^ as topdressing after the heading stage for both crops. The total amount of N, P and K was applied at 212, 25 and 47 kg ha^−1^ in wheat seasons, and at 281, 25 and 47 kg ha^−1^ in rice seasons, respectively. The basal fertilizer was broadcast and incorporated into the soil by ploughing to the depth of plow layer. Paddy rice was irrigated with the regimes of continuous flooding, with two periods of drainage in mid-season.

### Plant sampling and analysis

Plant samples were collected during the stem elongation, heading and ripening stages of rice in 2012 and of wheat in 2012–2013. The samples were separated into leaf, stem and ear. These samples were then oven-dried at 105 °C for 30 minutes and further oven-dried at 70 °C until constant weight. These oven-dried samples were then ground to yield a fine powder of <0.25 mm. Carbon concentration was measured with a CNS Macro Elemental Analyzer (Elementar, Germany). The plant leaf, stem and ear samples were pretreated with H_2_SO_4_-H_2_O_2_, dry-ashed at 550 °C for 4 h, and then the N concentration was determined with the Kjeldahl digestion method. Meanwhile, the P concentration (of acid digests) was analyzed by spectrophotometer (TU-1810, Beijing Purkinje General Instrument Co., Ltd., China).

### Statistical analysis

Statistical analyses were carried out in SPSS22.0 (IBM SPSS Statistics for Windows, Version 22.0. Armonk, NY, USA). We used a general linear mixed model (GLM) to test the main effects of elevated [CO_2_], warming and growth stage (main factor), and their interaction on nutrients uptake and their ratios. A post hoc test was followed if any treatment effect was significant (*p* < 0.05). All data were presented as mean plus or minus standard deviations.

## Supplementary information

Supporting Information
