# Supplementary material for: Changes in plant C, N and P ratios under elevated [CO2] and canopy warming in a rice-winter wheat rotation system
Source: Sci Rep. 2019 Apr 1;9:5424. doi: 10.1038/s41598-019-41944-1 (PMC6443658; doi:10.1038/s41598-019-41944-1)
Supplement: Supplementary file 1 — Supporting Information [file 41598_2019_41944_MOESM1_ESM.docx]

**Title:** **Changes in plant C, N and P ratios under elevated [CO_2_] and canopy warming in a rice-winter wheat rotation system**

Jianqing Wang ^1,2,3^, Xiaoyu Liu ^1,^*, Xuhui Zhang ^1^, Lianqing Li ^1^, Shu Kee Lam ^4^, Genxing Pan ^1^

^1^ Institute of Resource, Ecosystem and Environment of Agriculture, and Center of Climate Change and Agriculture, Nanjing Agricultural University, 1 Weigang, Nanjing 210095, China

^2^ Key Laboratory for Humid Subtropical Eco-geographical Processes of the Ministry of Education, Fujian Normal University, Fuzhou 350007, China

^3^ Tohoku Agricultural Research Center, National Agricultural and Food Research Organization, Iwate 020-0198, Japan

^4^ School of Agriculture and Food, Faculty of Veterinary and Agricultural Sciences, The University of Melbourne, Parkville, VIC 3010, Australia

*Corresponding author: Xiaoyu Liu

Email: [xiaoyuliu@njau.edu.cn](mailto:xiaoyuliu@njau.edu.cn)

Address: Institute of Resource, Ecosystem and Environment of Agriculture, and Center of Climate Change and Agriculture, Nanjing Agricultural University, 1 Weigang, Nanjing 210095, China.

Telephone: +86 25 8439 6127

Fax numbers: +86 25 84396507

Article Type: Research Paper

Running head: Effect of e[CO_2_] and warming on plant C, N and P ratios

**Table S1** Changes in C, N and P concentrations of rice under simulated climate change conditions.

| Growth stage | Treatment | Carbon g kg^-1^ | | | |  | Nitrogen g kg^-1^ | | | |  | Phosphorus g kg^-1^ | | | |
| --- | --- | --- | --- | --- | --- | --- | --- | --- | --- | --- | --- | --- | --- | --- | --- |
|  |  | Stem | Leaf | Panicle/Spike | Whole |  | Stem | Leaf | Panicle/Spike | Whole |  | Stem | Leaf | Panicle/Spike | Whole |
| Elongation | CK | 363.12b | 400.24b |  | 380.46b |  | 8.01ab | 11.82b |  | 9.79b |  | 4.11a | 2.66ab |  | 3.43a |
|  | CE | 374.37a | 408.43a |  | 391.07a |  | 6.92b | 8.75c |  | 7.83c |  | 3.20a | 2.29b |  | 2.75a |
|  | WA | 367.07ab | 407.95a |  | 385.92ab | | 9.65a | 15.40a |  | 12.3a |  | 3.56a | 3.09a |  | 2.99a |
|  | CW | 372.17a | 405.21ab | | 387.57a |  | 7.45ab | 11.79b |  | 9.47b |  | 3.60a | 2.60ab |  | 3.56a |
| Heading | CK | 375.17a | 405.20a | 379.55ab | 383.58a |  | 6.11a | 19.37b | 10.99ab | 10.23ab | | 2.55a | 3.12ab | 2.54b | 2.7b |
|  | CE | 374.6ab | 407.87a | 386.02a | 384.29a |  | 7.09a | 16.49c | 10.44bc | 9.79b |  | 2.18a | 2.65c | 2.76b | 2.35bc |
|  | WA | 371.30ab | 391.00a | 377.71ab | 377.86a |  | 6.02a | 23.60a | 9.78c | 11.44a |  | 2.32a | 3.32a | 3.31a | 3.42a |
|  | CW | 370.10b | 398.77a | 374.29b | 378.06a |  | 7.19a | 17.92bc | 11.98a | 10.63ab | | 2.42a | 3.03b | 3.50a | 2.23c |
| Ripening | CK | 374.19b | 383.89c | 384.67ab | 381.05b |  | 4.93a | 9.65b | 14.14b | 10.36a |  | 1.20a | 2.90a | 2.16b | 1.96a |
|  | CE | 383.02a | 400.50a | 394.17a | 390.61a |  | 6.39a | 7.53c | 13.86b | 9.8a |  | 0.85b | 2.71ab | 2.39ab | 1.81a |
|  | WA | 378.36b | 393.83ab | 373.53b | 379.03b |  | 5.98a | 11.64a | 15.62a | 10.71a |  | 0.85b | 2.13ab | 2.31ab | 1.75a |
|  | CW | 377.85b | 384.66bc | 388.90ab | 383.55ab | | 5.94a | 9.34b | 14.94ab | 10.4a |  | 1.04ab | 1.96b | 2.84a | 1.91a |
| [CO_2_] effect | | 1.03 | 0.98 | 1.84 | 1.19 |  | 0.41 | -21.47 | 1.32 | -10.68 |  | -8.90 | -11.26 | 11.97 | -9.994 |
| Warming effect | | -0.34 | -1.03 | -1.94 | -0.82 |  | 7.02 | 21.77 | 6.08 | 12.37 |  | -2.13 | -1.23 | 20.54 | 5.756 |
| [CO_2_] | | 0.001 | 0.089 | 0.030 | 0.001 |  | 0.937 | <0.001 | 0.495 | <0.001 |  | 0.070 | 0.012 | 0.010 | 0.017 |
| Warming | | 0.206 | 0.072 | 0.022 | 0.012 |  | 0.195 | <0.001 | 0.006 | <0.001 |  | 0.666 | 0.781 | <0.001 | 0.186 |
| Stage | | <0.001 | <0.001 | 0.061 | 0.004 |  | <0.001 | <0.001 | <0.001 | 0.044 |  | <0.001 | 0.001 | <0.001 | <0.001 |
| [CO_2_]×warming | | 0.012 | 0.024 | 0.738 | 0.048 |  | 0.252 | 0.132 | 0.023 | 0.452 |  | 0.013 | 0.999 | 0.586 | 0.265 |
| [CO_2_]×stage | | 0.004 | 0.898 | 0.082 | 0.062 |  | 0.008 | 0.107 | 0.015 | 0.001 |  | 0.359 | 0.589 | 0.486 | 0.010 |
| Warming×stage | | 0.116 | 0.050 | 0.810 | 0.053 |  | 0.430 | 0.336 | 0.040 | 0.016 |  | 0.952 | 0.001 | 0.048 | 0.387 |
| [CO_2_]×warming×stage | | 0.210 | 0.029 | 0.199 | 0.348 |  | 0.574 | 0.338 | 0.005 | 0.558 |  | 0.657 | 0.918 | 0.396 | 0.002 |

CK, ambient condition; CE, elevated [CO_2_] alone; WA, canopy warming alone; CW, elevated [CO_2_] and warming. Different letters indicate significant differences between treatments at *p* < 0.05.

**Table S2** Changes in C, N and P concentrations of wheat under simulated climate change conditions.

| Growth stage | Treatment | Carbon g kg^-1^ | | | |  | Nitrogen g kg^-1^ | | | |  | Phosphorus g kg^-1^ | | | |
| --- | --- | --- | --- | --- | --- | --- | --- | --- | --- | --- | --- | --- | --- | --- | --- |
|  |  | Stem | Leaf | Panicle/Spike | Whole |  | Stem | Leaf | Panicle/ Spike | Whole |  | Stem | Leaf | Panicle/Spike | Whole |
| Elongation | CK | 392.50a | 438.27a |  | 417.49a |  | 16.64b | 38.74bc |  | 28.70b |  | 4.03b | 3.67a |  | 3.84a |
|  | CE | 395.90a | 436.90a |  | 418.38a |  | 15.52bc | 36.07c |  | 26.75b |  | 4.68ab | 4.06a |  | 4.34a |
|  | WA | 383.33b | 421.70ab |  | 404.42b |  | 19.66a | 43.14a |  | 32.54a |  | 5.15ab | 3.98a |  | 4.51a |
|  | CW | 378.00b | 417.00b |  | 399.14b |  | 14.01c | 41.31ab |  | 28.83b |  | 5.83a | 3.97a |  | 4.82a |
| Heading | CK | 391.97a | 405.83b | 424.75a | 401.14a |  | 10.96a | 38.29a | 23.43a | 19.27a |  | 1.91b | 3.86ab | 3.912a | 2.71b |
|  | CE | 403.77a | 422.37a | 419.60a | 410.49a |  | 9.00a | 32.93bc | 21.53a | 16.21b |  | 1.35c | 2.57c | 4.47a | 2.17c |
|  | WA | 400.53a | 412.00ab | 420.47a | 406.94a |  | 9.23a | 35.89ab | 18.20b | 16.94b |  | 2.05b | 3.57b | 3.98a | 2.77b |
|  | CW | 402.20a | 422.63a | 420.35a | 410.06a |  | 10.36a | 32.56c | 22.03a | 17.38b |  | 2.62a | 4.25a | 4.46a | 3.32a |
| Ripening | CK | 401.03b | 413.30b | 402.27a | 402.65b |  | 2.46b | 20.92a | 18.19b | 13.61b |  | 1.61a | 1.41a | 4.01a | 3.11ab |
|  | CE | 427.43a | 430.70a | 400.27a | 411.05a |  | 1.66b | 19.26a | 16.52b | 11.98b |  | 1.33ab | 1.19ab | 3.66a | 2.74b |
|  | WA | 420.63a | 431.17a | 406.77a | 411.81a |  | 2.33b | 10.31b | 22.14a | 16.23a |  | 1.15b | 0.87b | 3.66a | 2.84ab |
|  | CW | 424.90a | 435.30a | 407.67a | 414.18a |  | 4.05a | 11.86b | 22.59a | 16.78a |  | 1.43ab | 0.83b | 4.56a | 3.46a |
| [CO_2_] effect | | 1.77 | 1.69 | -0.38 | 0.77 |  | -10.82 | -7.10 | 0.81 | -7.37 |  | 8.37 | -2.50 | 10.52 | 5.46 |
| Warming effect | | -0.12 | -0.30 | 0.51 | -0.59 |  | 5.98 | -5.99 | 6.70 | 10.45 |  | 22.82 | 4.17 | 3.53 | 14.81 |
| [CO_2_] | | 0.001 | 0.014 | 0.595 | 0.061 |  | 0.007 | <0.001 | 0.815 | <0.001 |  | 0.137 | 0.526 | 0.056 | 0.156 |
| Warming | | 0.792 | 0.642 | 0.487 | 0.141 |  | 0.147 | 0.001 | 0.075 | <0.001 |  | 0.001 | 0.309 | 0.485 | 0.001 |
| Stage | | <0.001 | 0.001 | <0.001 | 0.293 |  | <0.001 | <0.001 | 0.057 | <0.001 |  | <0.001 | <0.001 | 0.273 | <0.001 |
| [CO_2_]×warming | | 0.001 | 0.175 | 0.507 | 0.067 |  | 0.629 | 0.054 | 0.012 | 0.082 |  | 0.066 | 0.012 | 0.184 | 0.018 |
| [CO_2_]×stage | | 0.006 | 0.041 | 0.727 | 0.081 |  | 0.001 | 0.007 | 0.279 | 0.046 |  | 0.133 | 0.230 | 0.537 | 0.411 |
| Warming×stage | | <0.001 | <0.001 | 0.207 | <0.001 |  | 0.338 | <0.001 | <0.001 | <0.001 |  | 0.004 | 0.002 | 0.485 | 0.389 |
| [CO_2_]×warming×stage | | 0.296 | 0.738 | 0.856 | 0.999 |  | <0.001 | 0.620 | 0.216 | 0.017 |  | 0.337 | 0.001 | 0.104 | 0.078 |

CK, ambient condition; CE, elevated [CO_2_] alone; WA, canopy warming alone; CW, elevated [CO_2_] and warming. Different letters indicate significant differences between treatments at *p* < 0.05.

**Table S3** Changes in rice C, N and P ratios under simulated climate change conditions.

| Growth stage | Treatment | C:N | | | |  | C:P | | | |  | N:P | | | |
| --- | --- | --- | --- | --- | --- | --- | --- | --- | --- | --- | --- | --- | --- | --- | --- |
|  |  | Stem | Leaf | Panicle/Spike | Whole |  | Stem | Leaf | Panicle/Spike | Whole |  | Stem | Leaf | Panicle/Spike | Whole |
| Elongation | CK | 45.53ab | 34.3b |  | 38.92b |  | 88.6a | 150.45ab | | 110.89b |  | 1.95a | 4.44a |  | 2.86b |
|  | CE | 54.8a | 46.69a |  | 50.04a |  | 118.05a | 179.35a | | 142.47a | | 2.2a | 3.85a |  | 2.85b |
|  | WA | 38.17b | 26.5c |  | 31.38c |  | 105.87a | 136.85b | | 130.14ab | | 2.8a | 5.17a |  | 4.14a |
|  | CW | 52.4ab | 34.7b |  | 41.34b |  | 105.08a | 155.95ab | | 112.48b | | 2.09a | 4.54a |  | 2.77b |
| Heading | CK | 61.7a | 20.94b | 34.58ab | 37.51ab |  | 148.36a | 129.93bc | 150.49a | 142.63b | | 2.4a | 6.21ab | 4.38a | 3.8b |
|  | CE | 54.24a | 24.74a | 37.23a | 39.6a |  | 174.01a | 154.39a | 140.79a | 164.05a | | 3.3a | 6.24ab | 3.8ab | 4.19ab |
|  | WA | 61.92a | 16.64c | 38.63a | 33.08b |  | 160.61a | 117.82c | 114.46b | 111.23c | | 2.6a | 7.12a | 2.97b | 3.37b |
|  | CW | 52.09a | 22.42ab | 31.25b | 35.62ab |  | 155.05a | 131.65b | 106.89b | 169.53a | | 3.01a | 5.93b | 3.42b | 4.76a |
| Ripening | CK | 76.45a | 40.01bc | 27.29ab | 36.79ab |  | 319.77b | 134.01b | 178.25a | 195.22a | | 4.22a | 3.34bc | 6.56ab | 5.31a |
|  | CE | 60.96a | 53.19a | 28.46a | 39.89a |  | 458.89a | 148.83ab | 165.51ab | 215.63a | | 7.78a | 2.79c | 5.83ab | 5.41a |
|  | WA | 63.73a | 34.07c | 23.93c | 35.43b |  | 459.66a | 185.44ab | 161.97ab | 219.05a | | 7.32a | 5.5a | 6.77a | 6.22a |
|  | CW | 65.39a | 41.3b | 26.03bc | 37.09ab |  | 362.29ab | 214.88a | 140.6b | 207.53a | | 5.73a | 5.26ab | 5.38b | 5.55a |
| [CO_2_] effect | | -2.19 | 29.33 | -1.17 | 14.30 |  | 7.05 | 15.28 | -8.49 | 11.28 |  | 13.25 | -10.00 | -10.90 | -0.69 |
| Warming effect | | -5.65 | -20.12 | -6.05 | -11.86 |  | 3.13 | 5.09 | -17.50 | -2.16 |  | 7.69 | 24.73 | -9.79 | 9.73 |
| [CO_2_] | | 0.641 | <0.001 | 0.629 | <0.001 |  | 0.295 | 0.015 | 0.044 | 0.015 |  | 0.230 | 0.064 | 0.025 | 0.873 |
| Warming | | 0.228 | <0.001 | 0.019 | <0.001 |  | 0.633 | 0.368 | <0.001 | 0.599 |  | 0.470 | <0.001 | 0.043 | 0.040 |
| Stage | | 0.000 | <0.001 | <0.001 | 0.002 |  | <0.001 | 0.004 | <0.001 | <0.001 |  | <0.001 | <0.001 | <0.001 | <0.001 |
| [CO_2_]×warming | | 0.234 | 0.153 | 0.007 | 0.678 |  | 0.002 | 0.908 | 0.786 | 0.271 |  | 0.008 | 0.572 | 0.694 | 0.311 |
| [CO_2_]×stage | | 0.008 | 0.034 | 0.015 | 0.001 |  | 0.951 | 0.971 | 0.484 | 0.067 |  | 0.415 | 0.937 | 0.044 | 0.005 |
| Warming×stage | | 0.820 | 0.016 | 0.211 | 0.025 |  | 0.751 | 0.001 | 0.239 | 0.435 |  | 0.819 | 0.014 | 0.107 | 0.455 |
| [CO_2_]×warming×stage | | 0.343 | 0.204 | 0.002 | 0.888 |  | 0.008 | 0.782 | 0.653 | 0.031 |  | 0.038 | 0.495 | 0.081 | 0.037 |

CK, ambient condition; CE, elevated [CO_2_] alone; WA, canopy warming alone; CW, elevated [CO_2_] and warming. Different letters indicate significant differences between treatments at *p* < 0.05.

**Table S4** Changes in wheat C, N and P ratios under simulated climate change conditions.

| Growth stage | Treatment | C:N | | | |  | C:P | | | |  | N:P | | | |
| --- | --- | --- | --- | --- | --- | --- | --- | --- | --- | --- | --- | --- | --- | --- | --- |
|  |  | Stem | Leaf | Panicle/Spike | Whole |  | Stem | Leaf | Panicle/Spike | Whole |  | Stem | Leaf | Panicle/Spike | Whole |
| Elongation | CK | 23.59ab | 11.31b |  | 14.54b |  | 101.57a | 122.95a |  | 112.47a |  | 4.31a | 10.81a |  | 7.71a |
|  | CE | 25.65a | 12.12a |  | 15.64a |  | 85.1ab | 108.96a |  | 97.26ab |  | 3.35ab | 8.98a |  | 6.22a |
|  | WA | 19.5b | 9.8c |  | 12.44c |  | 75.03b | 105.98a |  | 89.83ab |  | 3.84a | 10.83a |  | 7.23a |
|  | CW | 27.45a | 10.1c |  | 13.88b |  | 65.22b | 105.83a |  | 83.31b |  | 2.4b | 10.47a |  | 6.00a |
| Heading | CK | 36.65a | 10.61c | 18.21b | 20.87b |  | 206.43b | 105.23bc | 109.32a | 148.46b |  | 5.74ab | 9.91b | 6.04a | 7.12a |
|  | CE | 44.97a | 12.84a | 19.49b | 25.33a |  | 304.74a | 164.32a | 94.06a | 189.61a |  | 6.78a | 12.81a | 4.83a | 7.48a |
|  | WA | 43.39a | 11.5b | 23.29a | 24.04a |  | 195.26b | 116.71b | 108.51a | 147.44b |  | 4.5bc | 10.2b | 4.74a | 6.14b |
|  | CW | 39.34a | 12.99a | 19.09b | 23.64a |  | 153.9b | 99.57c | 94.52a | 123.63c |  | 3.95c | 7.68c | 4.95a | 5.24c |
| Ripening | CK | 166.48ab | 19.77b | 22.13a | 29.59b |  | 255.43b | 304.72b | 101.15a | 130.73ab | | 1.59b | 15.52a | 4.58b | 4.42b |
|  | CE | 274.37a | 22.37b | 24.27a | 34.36a |  | 321.39ab | 377.03ab | 111.38a | 151.49a |  | 1.24b | 16.8a | 4.57b | 4.40b |
|  | WA | 197.46ab | 42.57a | 18.56b | 25.53c |  | 370.11a | 512.88a | 111.72a | 145.72ab | | 2.02ab | 11.97a | 6.08a | 5.75a |
|  | CW | 105.61b | 37.19a | 18.23b | 24.86c |  | 302.33ab | 534.49a | 90.25a | 120.50b |  | 2.85a | 14.51a | 4.95ab | 4.85ab |
| [CO_2_] effect | | 6.22 | 1.92 | -1.34 | 8.44 |  | 2.40 | 9.60 | -9.40 | -1.14 |  | -6.51 | 2.89 | -9.96 | -10.91 |
| Warming effect | | -24.30 | 39.45 | -5.87 | -11.37 |  | -8.85 | 24.70 | -2.63 | -14.41 |  | -15.01 | -12.26 | 3.48 | -5.71 |
| [CO_2_] | | 0.647 | 0.676 | 0.685 | <0.001 |  | 0.665 | 0.283 | 0.075 | 0.740 |  | 0.287 | 0.631 | 0.089 | 0.004 |
| Warming | | 0.044 | <0.001 | 0.083 | <0.001 |  | 0.100 | 0.014 | 0.615 | <0.001 |  | 0.015 | 0.036 | 0.563 | 0.114 |
| Stage | | <0.001 | <0.001 | 0.261 | <0.001 |  | <0.001 | <0.001 | 0.709 | <0.001 |  | <0.001 | <0.001 | 0.750 | <0.001 |
| [CO_2_]×warming | | 0.004 | 0.066 | 0.009 | 0.001 |  | <0.001 | 0.317 | 0.172 | 0.001 |  | 0.501 | 0.520 | 0.800 | 0.158 |
| [CO_2_]×stage | | 0.976 | 0.267 | 0.095 | 0.716 |  | 0.300 | 0.499 | 0.410 | 0.212 |  | 0.018 | 0.221 | 0.909 | 0.110 |
| Warming×stage | | 0.023 | <0.001 | <0.001 | <0.001 |  | <0.001 | <0.001 | 0.638 | 0.078 |  | <0.001 | 0.079 | 0.020 | <0.001 |
| [CO_2_]×warming×stage | | 0.001 | 0.116 | 0.276 | 0.025 |  | 0.018 | 0.597 | 0.141 | 0.006 |  | 0.052 | 0.087 | 0.046 | 0.349 |

CK, ambient condition; CE, elevated [CO_2_] alone; WA, canopy warming alone; CW, elevated [CO_2_] and warming. Different letters indicate significant differences between treatments at *p* < 0.05.

**Competing interests**

The authors declare no conflict of interest.
